# Assessing the relationship between monoallelic *PRKN* mutations and Parkinson’s risk

**DOI:** 10.1093/hmg/ddaa273

**Published:** 2021-01-15

**Authors:** Steven J Lubbe, Bernabe I Bustos, Jing Hu, Dimitri Krainc, Theresita Joseph, Jason Hehir, Manuela Tan, Weijia Zhang, Valentina Escott-Price, Nigel M Williams, Cornelis Blauwendraat, Andrew B Singleton, Huw R Morris, for International Parkinson’s Disease Genomics Consortium (IPDGC)

**Affiliations:** Ken and Ruth Davee Department of Neurology and Simpson Querrey Center for Neurogenetics, Feinberg School of Medicine, Northwestern University, Chicago, IL 60611, USA; Ken and Ruth Davee Department of Neurology and Simpson Querrey Center for Neurogenetics, Feinberg School of Medicine, Northwestern University, Chicago, IL 60611, USA; Ken and Ruth Davee Department of Neurology and Simpson Querrey Center for Neurogenetics, Feinberg School of Medicine, Northwestern University, Chicago, IL 60611, USA; Ken and Ruth Davee Department of Neurology and Simpson Querrey Center for Neurogenetics, Feinberg School of Medicine, Northwestern University, Chicago, IL 60611, USA; Department of Clinical and Movement Neurosciences, and UCL Movement Disorders Centre, Queen Square Institute of Neurology, University College London, London, WC1N 3BG, UK; National Hospital for Neurology and Neurosurgery, Queen Square, London, UK; Department of Clinical and Movement Neurosciences, and UCL Movement Disorders Centre, Queen Square Institute of Neurology, University College London, London, WC1N 3BG, UK; Department of Clinical and Movement Neurosciences, and UCL Movement Disorders Centre, Queen Square Institute of Neurology, University College London, London, WC1N 3BG, UK; Institute of Psychological Medicine and Clinical Neurosciences, Medical Research Council Centre for Neuropsychiatric Genetics and Genomics, Cardiff University School of Medicine, Cardiff, CF24 4HQ, UK; Dementia Research Institute at Cardiff, Cardiff University, Cardiff, CF24 4HQ, UK; Institute of Psychological Medicine and Clinical Neurosciences, Medical Research Council Centre for Neuropsychiatric Genetics and Genomics, Cardiff University School of Medicine, Cardiff, CF24 4HQ, UK; Molecular Genetics Section, Laboratory of Neurogenetics, National Institute on Aging, National Institutes of Health, Bethesda, MD 20892, USA; Molecular Genetics Section, Laboratory of Neurogenetics, National Institute on Aging, National Institutes of Health, Bethesda, MD 20892, USA; Department of Clinical and Movement Neurosciences, and UCL Movement Disorders Centre, Queen Square Institute of Neurology, University College London, London, WC1N 3BG, UK; See https://pdgenetics.org/partners

## Abstract

Biallelic Parkin (*PRKN*) mutations cause autosomal recessive Parkinson’s disease (PD); however, the role of monoallelic *PRKN* mutations as a risk factor for PD remains unclear. We investigated the role of single heterozygous *PRKN* mutations in three large independent case-control cohorts totalling 10 858 PD cases and 8328 controls. Overall, after exclusion of biallelic carriers, single *PRKN* mutations were more common in PD than controls conferring a >1.5-fold increase in the risk of PD [*P*-value (*P*) = 0.035], with meta-analysis (19 574 PD cases and 468 488 controls) confirming increased risk [Odds ratio (OR) = 1.65, *P* = 3.69E-07]. Carriers were shown to have significantly younger ages at the onset compared with non-carriers (NeuroX: 56.4 vs. 61.4 years; exome: 38.5 vs. 43.1 years). Stratifying by mutation type, we provide preliminary evidence for a more pathogenic risk profile for single *PRKN* copy number variant (CNV) carriers compared with single nucleotide variant carriers. Studies that did not assess biallelic *PRKN* mutations or consist of predominantly early-onset cases may be biasing these estimates, and removal of these resulted in a loss of association (OR = 1.23, *P* = 0.614; *n* = 4). Importantly, when we looked for additional CNVs in 30% of PD cases with apparent monoallellic *PRKN* mutations, we found that 44% had biallelic mutations, suggesting that previous estimates may be influenced by cryptic biallelic mutation status. While this study supports the association of single *PRKN* mutations with PD, it highlights confounding effects; therefore, caution is needed when interpreting current risk estimates. Together, we demonstrate that comprehensive assessment of biallelic mutation status is essential when elucidating PD risk associated with monoallelic *PRKN* mutations.

## Introduction

Parkinson’s disease (PD) is a multifactorial neurodegenerative disease. Common variations within 78 independent loci increase PD risk (1). Pathogenic mutations in autosomal dominant genes [leucine-rich repeat kinase 2 (*LRRK2*), α-synuclein (*SNCA*) and vacuolar protein sorting 35, yeast, homolog of (*VPS35*)] as well as biallelic mutations in autosomal recessive (AR) genes [*PRKN*, Parkinsonism associated deglycase (*PARK7*) or *DJ-1*, PTEN-induced putative kinase 1 (*PINK1*) and F-box protein 7 (*FBXO7*)] cause Mendelian PD (2). It has been suggested that single heterozygous pathogenic AR mutations can increase the risk of PD, and several lines of evidence have been provided for and against mutations (reviewed in Klein *et al*.) (3). Previous studies may have been confounded by the differences in methods for mutation detection in cases and controls. Biallelic AR mutations in PD genes are rare in PD cases, but single heterozygous mutations in specific AR PD genes are more common and are estimated, depending on the population, to occur in between 0.6 and 3% of unaffected control individuals (4–7). Accurate estimation of any risk associated with single heterozygous AR mutations is therefore essential for the counseling of biallelic carriers, monoallelic carriers and their family members. Furthermore, understanding the risk associated with single AR mutations may provide important insights into disease biology. Here, we investigate whether single carriers of disease-causing *PRKN* mutations are at an increased risk for PD using three large independent case-control cohorts using exome-focused genotype data, whole exome sequencing and resequencing (Reseq) data from the International Parkinson’s Disease Genomics Consortium (IPDGC).

**Table 1:** NeuroX Parkinson’s risk profiles associated with single heterozygous *PRKN* mutations

Type	*N* (Freq)		OR (95% CI)	*P*_log_	AAO (with data)	Coeff (95% CI)	*P*_reg_
	Controls	PD					
All							
With	37 (0.6%)	66 (1.0%)	1.55 (1.03, 2.32)	0.035	56.4 (86.4%)	−5.04 (−8.34, −1.75)	0.003
Without	5656	6486			61.4 (87.2%)		
CNV							
With	4 (0.1%)	11 (0.2%)	2.53 (0.80, 7.99)	0.113	58.4 (90.0%)	−3.11 (−11.15, 4.93)	0.449
Without	5689	6541			61.4 (87.2%)		
SNV							
With	33 (0.6%)	55 (0.8%)	1.43 (0.92, 2.21)	0.108	56.0 (85.5%)	−5.41 (−9.02, −1.81)	0.003
Without	5660	6497			61.4 (87.2%)		

Coeff, linear regression coefficient correcting for gender and principal components 1–4; Freq, frequency; *N*, number of samples; OR, odds ratio correcting for gender and principal components 1–4; *PRKN*, *Parkin* (NM_013988 and NM_004562); PD, Parkinson’s cases; *P*_log_, logistic regression *P*-value; *P*_reg_, linear regression *P*-value.

## Results

We identified a total of 109 monoallelic *PRKN* mutation carriers in 12 251 PD cases and controls (72 PD, 37 controls), carrying 19 different *PRKN* variants known to cause AR PD in the biallelic state, using the NeuroX genotyping platform (8). It is possible that the identified PD cases represent misclassified true biallelic *PRKN* PD cases. To confirm whether PD cases carry a single pathogenic allele or whether a second variant was missed, we (i) reviewed diagnostic reports if available (*n* = 4) or (ii) assessed available samples using multiplex ligation-dependent probe amplification (MLPA, *n* = 29). Of the 33 available NeuroX samples, representing ~30% of our putative monoallelic individuals [five controls, 13.5%; 28 PD cases harbouring a single heterozygous *PKRN* mutation (PD-monoallelic), 38.9%], six cases (18% of the available samples, 21% of available PD cases) were found to harbour a second mutation and therefore were removed, leaving a total of 66 PD cases for all subsequent analyses (no controls were found to harbour a second mutation).

After removal of cases with established Mendelian mutations across all known PD genes, 1.0% (66/6552) of PD cases were found to harbour single heterozygous *PRKN* PD-causing mutations [either heterozygous copy number variants (CNVs) or single nucleotide variants (SNVs), Table 1], compared with 0.6% (37/5693) of controls. Single heterozygote mutations might increase the PD risk [Odds ratio (OR) = 1.55; 95% confidence interval (CI):1.03, 2.33; *P*-value (*P*) = 0.035], although ~70% of putative monogenic cases were not assessed for a second mutation. Although speculative, if the remaining apparent monoallelic cases had a similar rate of occult biallelic mutations, then the true underlying monoallelic carrier rate could be estimated to be lower at 0.9%, and there would be no difference between cases and controls (OR = 1.39; 95% CI: 0.90, 2.16; *P* = 0.117). Using age at onset (AAO) data from 5710 (87.1%) cases, we found that NeuroX PD cases with single *PRKN* mutations have significantly lower AAOs (average = 56.4 years) than cases without known mutations [average = 61.4 years; β-coefficient (Coeff) = −5.04; 95% CI: −8.32, −1.71; *P* = 0.003].

**Figure 1:**
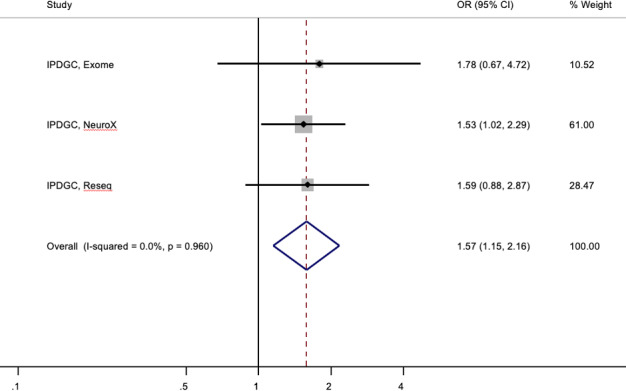
Forest plot of OR of the Parkinson’s risk associated with heterozygous *PRKN* mutations in three independent IPDGC cohorts.

We next sought to explore the potential increased risk in two independent IPDGC case-control cohorts using exome sequencing (cases = 1235; controls = 473) (8) and Reseq data (cases = 3071; controls = 2162). We identified 28 (23 cases, five controls) and 52 (36 cases, 16 controls) carriers of single *PRKN* mutations in the exome [as previously described (8)] and Reseq data, respectively. CNVs were not determined in the primary exome or Reseq dataset. In the exome cohort, 1.9% (23/1231) of cases and 1.1% (5/473) of controls, and in the Reseq cohort, 1.2% (36/3071) of cases and 0.7% (16/2162) of controls harboured single *PRKN* SNVs. Before searching for occult second mutations, a meta-analysis of the three IPDGC (NeuroX, exome sequencing and Reseq) cohorts revealed a significant ~1.5-fold increased risk [OR = 1.57; 95% CI: 1.15, 2.16; *P* = 0.005; proportion of total variation caused by heterogeneity (*I*^2^) = 0.0%, Cochran’s Q-statistic test for heterogeneity *P*-value (*P*_het_) = 0.960] associated with *PRKN* mutations (Fig. 1). AAO data was available on 1130 PD exome cases (91.8%) and 2599 Reseq cases (84.6%). Albeit non-significant, exome PD cases carrying single *PRKN* SNVs had lower AAO compared with non-carriers (average = 38.5 years vs. 43.1 years; Coeff = −4.34; 95% CI: −8.95, 0.28; *P* = 0.066), with carriers having significantly lower AAO in Reseq cases (average = 52.6 years vs. 60.5 years; Coeff = −7.84; 95% CI: −12.59, −3.09; *P* = 0.001). We then used MLPA to search for potentially missed *PRKN* CNVs in mutation carriers. Four of the nine available exome deoxyribonucleic acid (DNA) samples (44%, all PD cases) were found to harbour a missed second mutation and were removed from the subsequent analyses. Assuming a similar rate of occult biallelic carriage across both datasets, the true rate of monoallelic cases could be estimated to be 1.2 and 0.7% in the exome and Reseq cases as compared with the 1.1 and 0.7% of the controls, respectively.

**Figure 2:**
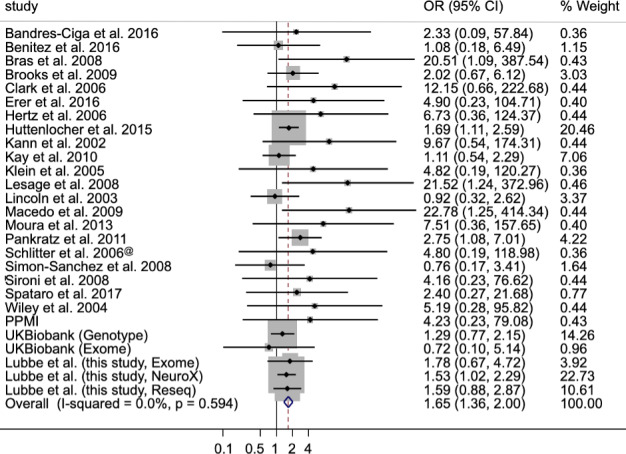
Forest plot of the OR of the Parkinson’s risk associated with heterozygous *PRKN* mutations.

We next performed a meta-analysis of available cohorts and studies that reported heterozygous PD-causing mutation rates in cases and controls, from European ancestry cohorts only. Three cohorts (Parkinson’s Progression Markers Initiative, PPMI, https://www.ppmi-info.org/; UK Biobank Genotyping and Exome cohorts, https://www.ukbiobank.ac.uk/) and 21 published studies were included in our analyses (5,6,9–27). Including our cohorts, the meta-analysis revealed a significant 1.65-fold increased PD risk in single *PRKN* mutation carriers (95% CI: 1.36, 2.00; *P* = 3.69E-07; *I*^2^ = 0.0%, *P*_het_ = 0.594) (Fig. 2).

As in our study, occult second mutations are likely biasing these estimates, so we therefore restricted our meta-analysis to nine studies (9/21, 43%) that searched for a second *PRKN* mutation (Table 2). Based on these studies, single *PRKN* mutations confer a 2-fold increase in PD risk in carriers (OR = 2.00, 95% CI: 1.10, 3.62, *P* = 0.023; *I*^2^ = 1.3%, *P*_het_ = 0.423) (Supplementary Material, Fig. S1).

**Table 2:** Meta-analyses of heterozygous *PRKN* mutation carriers in studies that investigated and excluded PD patients with biallelic *PRKN* mutations

Study	PD		Controls		OR (95% CI)
	Carrier	Non-carrier	Carrier	Non-carrier	
Benitez *et al*. 2016	3	466	2	335	1.08 (0.18–6.49)
Brooks *et al*. 2009	9	241	5	271	2.02 (0.67–6.12)
Clark *et al*. 2006	5	95	0	105	12.15 (0.66–222.68)
Hertz *et al*. 2006	5	82	0	50	6.73 (0.37–124.37)
Klein *et al*. 2005	1	62	0	100	4.82 (0.19–120.27)
Lesage *et al*. 2008	9	150	0	170	21.35 (1.24–372.96)
Lincoln *et al*. 2003	9	304	6	186	0.92 (0.32–2.62)
Spataro *et al*. 2017	4	240	1	144	2.40 (0.27–21.68)
Wiley *et al*. 2004	5	96	0	45	5.19 (0.28–95.82)
Pooled	50	1736	9	1406	2.00 (1.10–3.62)

Carrier, number of samples harbouring mutation, *PRKN*, *Parkin* (NM_013988 and NM_004562).

Inclusion of studies that used predominantly early-onset PD (EOPD) cases may additionally be inflating these estimates; we therefore repeated the meta-analysis excluding these EOPD studies (15/21, 71%), which demonstrated a 1.5-fold significant increased risk in carriers (OR = 1.50, 95% CI: 1.22, 1.84; *P* = 1.05E-04; *I*^2^ = 0%, *P*_het_ = 0.927) (Supplementary Material, Fig. S2).

Restricting our analysis to the four non-EOPD studies, that searched for biallelic carriers, demonstrated that single *PRKN* mutations were not associated with an increased PD risk in these cohorts (OR = 1.23, 95% CI: 0.55, 2.75, *P* = 0.614; *I*^2^ = 0.0%, *P*_het_ = 0.657; Supplementary Material, Fig. S3).

The pathogenicity of the common *PRKN* p.R275W variant in AR PD is not as clear cut as other *PRKN* mutations. To test whether the observed SNV association is driven by p.R275W, we repeated the meta-analysis after excluding this variant. Removal of p.R275W resulted in a marginally increased estimate (OR = 1.76, 95% CI: 1.37, 2.28, *P* = 1.42E-05; *I*^2^ = 0.0%, *P*_het_ = 0.673; Supplementary Material, Table S1; Fig. S4). Limiting our analysis to the eight cohorts which assessed biallelic mutations indicated a >2-fold increased risk (OR = 2.41, 95% CI: 1.17, 4.96, *P* = 0.017; *I*^2^ = 0.0%, *P*_het_ = 0.791) (Supplementary Material, Fig. S5).

The contribution of biallelic *PRKN* CNVs to AR PD is well established; however, that of heterozygous CNV carriers remains unclear. We identified monoallelic *PRKN* CNVs in 0.17% (11/6552) of non-Mendelian PD cases compared with 0.07% (4/5693) controls (Table 1) using the NeuroX data only. None of these CNV carriers overlapped with NeuroX SNV carriers. There was a >2.5-fold increase in PD risk for *PRKN* CNV heterozygote carriers compared with controls (OR = 2.53; 95% CI: 0.80, 7.99; *P* = 0.113), but this was not statistically significant.

It has been suggested that monoallelic *PRKN* CNVs might confer a higher risk that is associated with a more pathogenic profile compared with other AR mutations (28). To assess this, we compared differences in risk between CNV and SNVs carriers in the NeuroX cohort. A total of 55 *PRKN* SNV carriers were seen in non-Mendelian PD cases (55/6552; 0.8%) compared with 33 controls (33/5693; 0.6%) (OR = 1.43; 95% CI: 0.92, 2.21; *P* = 0.108) (Table 1). To test whether *PRKN* CNVs confer a more ‘pathogenic’ risk profile compared with SNVs, we performed AAO analysis in the NeuroX data only. Carriers of heterozygous *PRKN* CNVs had a mean AAO of 58.4 years, compared with non-carriers (61.4 years) (Coeff = −3.11; 95% CI: −11.15, 4.93; *P* = 0.449).

**Figure 3:**
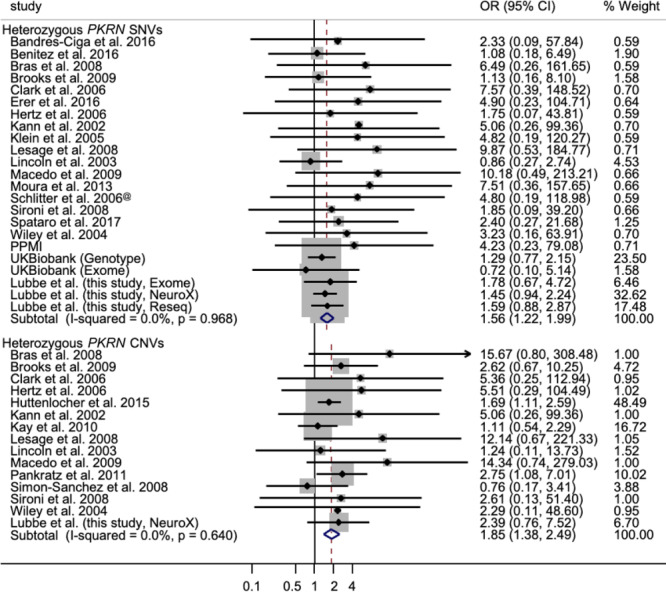
Forest plot of the OR of the Parkinson’s risk associated with heterozygous *PRKN* SNV and CNV carriers.

To further investigate the potential different risk profiles, we performed separate meta-analyses of published *PRKN* mutation data for SNVs and CNVs. Meta-analyses, including the current data, revealed significant independent increased PD risks for SNVs (OR = 1.56, 95% CI: 1.22, 2.00, *P* = 4.46E-04; *I*^2^ = 0%, *P*_het_ = 0.968) and CNVs (OR = 1.85, 95% CI: 1.38, 2.50, *P* = 4.55E-05; *I*^2^ = 0.0%, *P*_het_ = 0.640) (Fig. 3; Supplementary Material, Table S2). Restricting our meta-analysis to studies that searched for second hits suggested that PD risk was larger in the carriers of single *PRKN* CNVs (OR = 3.11, 95% CI: 1.23, 7.89, *P* = 0.016; *I*^2^ = 0.0%, *P*_het_ = 0.879) compared with those harbouring heterozygous SNVs (OR = 1.59, 95% CI: 0.79, 3.20, *P* = 0.191; *I*^2^ = 0.0%, *P*_het_ = 0.785).

## Discussion

The role of rare biallelic mutations in *PRKN* in AR PD (MIM#600116) is well established. Here, using data from a large PD case-control cohort, we identified a total of 109 carriers of single heterozygous *PRKN* mutations. After exclusion of PD cases with known mutations, we demonstrated that carriers of single mutations were at a small but significantly increased risk of PD (OR = 1.55; 95% CI: 1.03, 2.33; *P* = 0.035). This was confirmed by a meta-analysis with two additional IPDGC cohorts (cases = 10 954; controls = 8328) which demonstrated a significant >1.5-fold increased risk (*P* = 0.005). Carriers also had significantly lower AAOs than non-carriers (56.4 years vs. 61.4 years; *P* = 0.003). Similar findings were seen in the exome and Reseq data for increased risk [exome, OR = 2.20; Reseq, OR = 1.59] and younger AAOs compared with non-carriers (exome, 38.5 years vs. 43.1 years; Reseq, 52.6 years vs. 60.5 years). A meta-analysis of 19 574 PD cases and 468 488 controls from 27 cohorts further confirmed that heterozygous *PRKN* mutations confer an increased PD risk (OR = 1.65; *P* = 3.69E-07). However, several confounding factors are likely biasing these estimates in favour of increased risk and are explored in this study. Large-scale studies in systematically recruited cohorts that have comprehensively interrogated biallelic *PRKN* mutations are therefore needed to accurately determine the risk associated with single mutations.

The relatively common p.R275W (c.823C > T, rs34424986) variant, the most frequent PD-associated variant in *PRKN*, has not been reported in the homozygous state and has only been reported in compound heterozygotes with another mutation in multiple AR PD families (MIM#602544), and it has been classified as likely pathogenic. p.R275W reduces protein stability by disrupting binding to phosphorylated ubiquitin and results in reduced Parkin (*PRKN*) levels (29), supporting the pathogenicity of p.R275W. We examined whether the increased PD risk associated with single *PRKN* variants was driven by this variant. The observation that the OR increases after removal suggests that p.R275W may have reduced effect on enzyme activity compared with other mutations, and that, because of its more common frequency, its presence may be diluting the true effect of heterozygous *PRKN* mutations in PD biology.

Our analysis provides some support for a more ‘pathogenic’ risk profile associated with *PRKN* CNVs as compared with SNVs (CNVs, OR = 2.53; SNVs, OR = 1.43) in the NeuroX data. Both mutation types appear to be associated with lower AAOs; however, the small number of observed CNVs prevents any definitive conclusions from being drawn. While the meta-analysis results here support the increased risk for *PRKN* CNV carriers (OR = 1.85), albeit marginally higher than *PRKN* SNVs (OR = 1.56), the increased risk associated with *PRKN* CNVs should be interpreted with care. Small sample sizes, low CNV frequency and failure to investigate/report CNVs may have resulted in an underestimated effect size seen in the meta-analysis. Failure to conclusively look for second *PRKN* hits may also be a potential confounder when trying to estimate the risk associated with single *PRKN* mutations. Additional work in larger cohorts where both *PRKN* SNVs and CNVs are routinely assessed is therefore needed to gain more accurate insight into the different risk profiles associated with different mutation types.

This large study builds on previous work looking at single *PRKN* mutations in PD aetiology. While several studies failed to identify single known pathogenic *PRKN* mutations in controls thereby supporting increased disease risk, others have found equal frequencies in both cases and controls providing evidence against increased risk (4,7,13,17,18,30–32). These estimates have, however, been based on relatively small sample sets which have made it difficult to conclusively determine if single mutations confer any risk. The inclusion of non-ClinVar (https://www.ncbi.nlm.nih.gov/clinvar/) variants represents a potential confounder in that we may be overestimating the frequency of disease relevant single *PRKN* mutations. Limiting our analyses to ClinVar variants only did not result in considerably different risk estimates across all comparisons (all studies, OR = 1.70, *P* = 2.65E-07; biallelic studies, OR = 1.99, *P* = 0.036; non-EOPD studies, OR = 1.55, *P* = 5.6E-05). Another confounder relates to the fact that we observed a significant rate of occult second pathogenic mutations in putative monoallelic cases in our NeuroX cohort. The detected rate of occult biallelic carrier status was high in our two datasets (6/28, 21% and 4/9, 44%), approaching one-half of PD cases with apparent monoallelic status. Additionally, several studies included in the analyses here have not searched for potentially hidden biallelic mutations in all cases and controls or have only interrogated a subset of *PRKN* mutations. Inclusion of these PD cases in our analysis is likely to appreciably influence our estimate. However, restricting the meta-analyses to nine cohorts that searched for biallelic *PRKN* mutations in all cases and controls, demonstrated that single mutations confer a 2-fold increase in risk in carriers. A further confounding factor is the use of EOPD cases (<50 years) in such studies which may be additionally inflating risk estimates as *PRKN* mutations are more likely to occur in PD cases of younger onset. This was observed in the IPDGC cohorts, with a higher estimate in the exome cohort compared with the NeuroX and Reseq cohorts. The additional removal of predominantly EOPD studies resulted in the loss of the original association (OR = 1.23; *P* = 0.614), but this was based on a few small studies (*n* = 4). This suggests that the current estimates of the effect of single mutations in modulating PD risk may not be accurate but also stresses the importance of comprehensively searching for biallelic mutations in systematically recruited cohorts. It remains possible that there are further ‘occult’ coding variants of unknown significance or non-coding mutations affecting the promoter or splicing regions that have not yet been identified.

There are some limitations to our study. NeuroX biallelic and monoallelic cases will have been missed as not all possible PD-causing variants are represented on the chip, with only 16.8% of known pathogenic variants present (33) (Supplementary Material, Table S3). The same applies to the detection of biallelic carriers in the UK Biobank genotyping cohort. Identifying *PRKN* CNVs from the NeuroX variant genotype data using PennCNV may have missed smaller deletions/duplications. The false-positive rate of PennCNV as a method for CNV detection was estimated to be 9.0–17.7%, with false-positive CNVs predominantly small in size and occurring regardless of genotyping chip used (34). Our CNV detection of false positive rate in the NeuroX cohort is 6.9%. However, the fact that (i) the NeuroX variants are not evenly distributed across the *PRKN* locus (accounts for four misclassified samples) and that (ii) we were looking for CNVs as small as a single exon may have resulted in our approach missing or inaccurately calling CNVs in our large cohort comprising predominantly of late-onset PD cases. There are limitations in defining CNVs from the IPDGC and UK Biobank exome data, so CNVs were only investigated using MLPA in identified PD-monoallelic exome cases. As the exome cohort predominantly consists of EOPD cases, it is likely that additional *PRKN* CNVs carriers were undetected. We therefore sought to validate the monoallelic status of the available carriers by accessing the diagnostic reports or by directly assessing CNVs using MLPA and discovered a high rate of undetected second hits in both our datasets. Previous studies which have not systematically searched for second hits may have therefore erroneously determined the monoallelic carrier rate, meaning that the estimates derived from our in-house cohorts and other published meta-analyses may not be accurate. It is therefore very important that any proposed increased risk associated with single *PRKN* mutations be considered with caution as, based on findings presented here, a substantial part of the reported excess on monoallelic carriers may relate to occult biallelic status.

Across AR diseases, there is a great deal of interest in the potential role of single heterozygous mutations as risk factors for disease. Heterozygous *PINK1* mutations in PD (35) and *MUTYH* mutations in colorectal cancer (36) do not confer an increased risk of PD or cancer, respectively. However, single AR mutations may increase the risk of related, but separate conditions from the prototypic recessive disease. Single *CFTR* mutation carriers are more susceptible to cystic fibrosis-related conditions (37), and monoallelic *ATM* mutation carriers have higher risks for cancers and ischemic heart disease—especially breast cancer in female carriers. This current study highlights the importance of ensuring that all potential confounders are taken into consideration when assessing single mutations as any unaccounted-for biases would generate inaccurate risk estimates and have significant repercussions on the counseling of patients and their family members.

In conclusion, while much of the data demonstrate that harbouring a single heterozygous *PRKN* mutation increases PD risk and that single *PRKN* CNVs may be more pathogenic than *PRKN* SNVs, there may be confounding factors. This is supported by our finding of no increased risk associated with single *PRKN* mutations upon restricting our analysis to studies that assessed biallelic mutations in cases and controls and studies that did not include predominantly EOPD cases. Before the risk associated with single heterozygous mutations can be accurately defined, we highlight the importance of assessing ‘second hits’ in all cases and controls, where both SNVs and CNVs are systematically interrogated in large-scale cohorts that have been systematically recruited.

## Materials and Methods

High-quality genotype data from the NeuroX chip on 6558 PD cases and 5693 controls were assessed as part of the IPDGC [Database of Genotypes and Phenotypes (dbGaP) Study Accession number: phs000918.v1.p1] (see Supplementary Material, Table S4 for full list of *PRKN* variants captured). Sample collection and variant genotyping have been described elsewhere (33). IPDGC exome sequencing data from 1235 PD cases and 473 controls were used as a replication cohort [European Genome-phenome Archive (EGA) Study Accession numbers: EGAS00001002103, EGAS00001002110, EGAS00001002113, EGAS00001002156; dbGaP Study Accession number: phs001103.v1.p1] and are described elsewhere (8). Additional replication cohorts used include: IPDGC Reseq cohort (cases = 3071, controls = 2162), UK Biobank Genotyping (cases = 1428, controls = 312 098; downloaded April 2018, under application number 33601) and UK Biobank exome sequencing (cases = 114, controls = 38 263; downloaded May 2019) cohorts and the PPMI Exome sequencing *de novo* cohort (cases = 385, controls = 179). The IPDGC Reseq cohort (PD: 54.8% male, average AAO = 63.8 years; controls: 44.8% male, average age at recruitment = 60.4 years) targeted sequencing genetic data covering PD-associated genes [including *ATP13A2*, *FBXO7*, *GBA*, *LRRK2*, *MAPT*, *PARK7* or *DJ-1*, *PINK1*, *PLA2G6*, *PRKN*, *SNCA* and *VPS35*] (35,38). Duplicate samples were removed, where possible, from all analyses. Samples with missing call rates >5% were excluded during quality control. Variants (excluding synonymous) from known Mendelian PD-causing genes were extracted. Pathogenic mutations were identified as previously described (8). Rare *PRKN* (NM_013988 and NM_004562) CNVs were identified in the NeuroX cohort using PennCNV (34). CNVs spanning a minimum of 10 variants were selected and visually confirmed. Monoallelic PD cases were defined as those carrying a single heterozygous pathogenic *PRKN* allele as defined according to the Online Mendelian Inheritance in Man (OMIM) (http://omim.org/), the Movement Disorder Society Genetic mutation database (https://www.mdsgene.org/) or the Parkinson Disease Mutation Database (http://www.molgen.vib-ua.be/PDMutDB/) (Supplementary Material, Table S4). Where available, samples were investigated by (i) accessing sample diagnostic records or by (ii) using MLPA [SALSA P051 v.D1 probe mix (Microbiology Research Centre Holland, MRC-Holland, The Netherlands)] to confirm their monoallelic status. Without phasing information, any two PD-causing hits identified in an individual are assumed to be in *trans*.

To assess whether the PD risk might be associated with (i) all monoallelic variants, (ii) CNVs alone or (iii) SNVs alone, as indicated by case-control differences, we used logistic regression correcting for gender and principal components 1–4 (C1–4). Linear regression was used to investigate the impact of single AR mutations on AAO.

A literature review was undertaken (on 1 October 2019) to identify published data on heterozygous *PRKN* mutations, using search terms including combinations of the following terms: Parkinson’s disease, PD, *PRKN*, *PARK2* and heterozygous. Additional studies were identified by manual search of references cited in published articles. Should any of the studies include previously published data, the most recent data were selected where possible. Meta-analysis was conducted using standard methods modeling fixed effects, using Cochran’s Q-statistic to test for heterogeneity (*P*_het_) (39) and the *I*^2^ statistic (40) to quantify the proportion of the total variation caused by heterogeneity relating to possible differences in sample recruitment and assessment between studies. Meta-analyses were performed for CNVs and SNVs separately to investigate potential different risk profiles for each mutation type.

## Supplementary Material

2020_12_09_Monoallelic_v31_HMG_suppl_data_accepted_ddaa273
