# Supplementary material for: Assessing the relationship between monoallelic PRKN mutations and Parkinson’s risk
Source: Hum Mol Genet. 2021 Jan 15;30(1):78–86. doi: 10.1093/hmg/ddaa273 (PMC8033143; doi:10.1093/hmg/ddaa273)
Supplement: 2020_12_09_Monoallelic_v31_HMG_suppl_data_accepted_ddaa273 [file 2020_12_09_monoallelic_v31_hmg_suppl_data_accepted_ddaa273.docx]

**ASSESSING THE RELATIONSHIP BETWEEN MONOALLELIC *PRKN* MUTATIONS AND PARKINSON’S RISK: SUPPLEMENTARY INFORMATION**

Supplementary Table 1: Meta-analyses of heterozygous *PRKN* variant carriers, excluding R275W (c.823C>T, rs34424986).

| Study | PD | | Controls | | OR (95% CI) |
| --- | --- | --- | --- | --- | --- |
|  | Carrier | Non-carrier | Carrier | Non-carrier |  |
| Bandres-Ciga et al. 2016 | 1 | 125 | 0 | 97 | 2.33 (0.09-57.84) |
| Benitez et al. 2016 | 1 | 468 | 0 | 337 | 2.16 (0.09-53.21) |
| Bras et al. 2008 | 4 | 55 | 0 | 126 | 20.51 (1.09-387.54) |
| Brooks et al. 2009 | 8 | 242 | 4 | 272 | 2.25 (0.67-7.56) |
| Clark et al. 2006 | 4 | 96 | 0 | 105 | 9.84 (0.54-185.15) |
| Erer et al. 2016 | 2 | 48 | 0 | 47 | 4.90 (0.23-104.71) |
| Hertz et al. 2006 | 5 | 82 | 0 | 50 | 6.73 (0.37-124.37) |
| Huttenlocher et al. 2015 | 22 | 1,391 | 965 | 103,355 | 1.69 (1.11-2.59) |
| Kann et al. 2002 | 6 | 101 | 0 | 75 | 9.67 (0.54-174.31) |
| Kay et al. 2010 | 17 | 1,920 | 13 | 1,629 | 1.11 (0.54-2.29) |
| Lesage et al. 2008 | 5 | 154 | 0 | 170 | 12.14 (0.67-221.33) |
| Lincoln et al. 2003 | 5 | 306 | 3 | 189 | 1.03 (0.24-4.36) |
| Macedo et al. 2009 | 5 | 175 | 0 | 363 | 22.78 (1.25-414.34) |
| Moura et al. 2013 | 2 | 133 | 0 | 200 | 7.51 (0.36-157.65) |
| Pankratz et al. 2011 | 10 | 386 | 8 | 848 | 2.75 (1.08-7.01) |
| Schlitter et al. 2006^*^ | 1 | 93 | 0 | 149 | 4.80 (0.19-118.98) |
| Simon-Sanchez et al. 2008 | 3 | 269 | 4 | 271 | 0.76 (0.17-3.41) |
| Sironi et al. 2008 | 5 | 133 | 0 | 50 | 4.16 (0.23-76.62) |
| Spataro et al. 2017 | 10 | 232 | 1 | 144 | 2.40 (0.27-21.68) |
| Wiley et al. 2004 | 2 | 99 | 0 | 45 | 2.29 (0.11-48.60) |
| PPMI | 1 | 384 | 0 | 179 | 1.40 (0.06-34.55) |
| UKBiobank (Exome) | 1 | 113 | 140 | 38,123 | 2.41 (0.33-17.38) |
| Lubbe et al. (IPDGC, Exome) | 7 | 1,224 | 1 | 472 | 2.70 (0.33-21.99) |
| Lubbe et al. (IPDGC, NeuroX) | 17 | 6,535 | 15 | 5,678 | 0.99 (0.49-1.97) |
| Lubbe et al. (IPDGC, Reseq) | 12 | 3,059 | 4 | 2,158 | 2.12 (0.68-6.57) |
| **Pooled** | **150** | **17,831** | **1,158** | **155,132** | **1.76 (1.37-2.28)** |

Key: CI, confidence intervals; IPDGC, International Parkinson’s disease Genomics Consortium; *PRKN*, *Parkin* (NM_013988 and NM_004562); OR, odds ratio; PPMI, Parkinson’s Progression Markers Initiative; Reseq, Resequencing; *German samples only.

Supplementary Table 2: Meta-analyses of heterozygous *PRKN* mutation carriers, stratified by type of mutation.

|  | Study | PD | | Controls | | OR (95% CI) | |
| --- | --- | --- | --- | --- | --- | --- | --- |
|  |  | Carrier | Non-carrier | Carrier | Non-carrier |  | |
| Heterozygous *PRKN* SNVs | |  |  |  |  | |  |
|  | Bandres-Ciga et al. 2016 | 1 | 125 | 0 | 97 | 2.33 (0.09-57.84) | |
|  | Benitez et al. 2016 | 3 | 466 | 2 | 335 | 1.08 (0.18-6.49) | |
|  | Bras et al. 2008 | 1 | 58 | 0 | 126 | 6.49 (0.26-161.65) | |
|  | Brooks et al. 2009 | 2 | 242 | 2 | 274 | 1.13 (0.16-8.10) | |
|  | Clark et al. 2006 | 3 | 97 | 0 | 105 | 7.57 (0.39-148.52) | |
|  | Erer et al. 2016 | 2 | 48 | 0 | 47 | 4.90 (0.23-104.71) | |
|  | Hertz et al. 2006 | 1 | 86 | 0 | 50 | 1.75 (0.07-43.81) | |
|  | Kann et al. 2002 | 3 | 104 | 0 | 75 | 5.06 (0.26-99.36) | |
|  | Klein et al. 2005 | 1 | 62 | 0 | 100 | 4.82 (0.19-120.27) | |
|  | Lesage et al. 2008 | 4 | 155 | 0 | 170 | 9.87 (0.53-184.77) | |
|  | Lincoln et al. 2003 | 7 | 305 | 5 | 187 | 0.86 (0.27-2.74) | |
|  | Macedo et al. 2009 | 2 | 178 | 0 | 363 | 10.18 (0.49-213.21) | |
|  | Moura et al. 2013 | 2 | 133 | 0 | 200 | 7.51 (0.36-157.65) | |
|  | Schlitter et al. 2006* | 1 | 93 | 0 | 149 | 4.80 (0.19-118.98) | |
|  | Sironi et al. 2008 | 2 | 136 | 0 | 50 | 1.85 (0.09-39.20) | |
|  | Spataro et al. 2017 | 4 | 240 | 1 | 144 | 2.40 (0.27-21.68) | |
|  | Wiley et al. 2004 | 3 | 98 | 0 | 45 | 3.23 (0.16-63.91) | |
|  | PPMI | 4 | 381 | 0 | 179 | 4.24 (0.23-79.08) | |
|  | UKBiobank (Genotype) | 15 | 1,413 | 2,548 | 309,550 | 1.29 (0.77-2.15) | |
|  | UKBiobank (Exome) | 1 | 113 | 467 | 37,796 | 0.72 (0.10-5.14) | |
|  | Lubbe et al. (IPDGC, Exome) | 23 | 1,208 | 5 | 468 | 1.78 (0.67-4.72) | |
|  | Lubbe et al. (IPDGC, NeuroX) | 55 | 6,497 | 33 | 5,660 | 1.45 (0.94-2.24) | |
|  | Lubbe et al. (IPDGC, Reseq) | 36 | 3,035 | 16 | 2,146 | 1.59 (0.88-2.87) | |
|  | **Pooled** | **176** | **15,273** | **3,079** | **358,316** | **1.56 (1.22-2.00)** | |
| Heterozygous *PRKN* CNVs | |  |  |  |  | |  |
|  | Bras et al. 2008 | 3 | 56 | 0 | 126 | 15.67 (0.80-308.48) | |
|  | Brooks et al. 2009 | 7 | 243 | 3 | 273 | 2.62 (0.67-10.25) | |
|  | Clark et al. 2006 | 2 | 98 | 0 | 105 | 5.36 (0.25-112.94) | |
|  | Hertz et al. 2006 | 4 | 82 | 0 | 50 | 5.51 (0.29-104.49) | |
|  | Huttenlocher et al. 2015 | 22 | 1,391 | 965 | 103,355 | 1.69 (1.11-2.59) | |
|  | Kann et al. 2002 | 3 | 104 | 0 | 75 | 5.06 (0.26-99.36) | |
|  | Kay et al. 2010 | 17 | 1,920 | 13 | 1,629 | 1.11 (0.54-2.29) | |
|  | Lesage et al. 2008 | 5 | 154 | 0 | 170 | 12.14 (0.67-221.33) | |
|  | Lincoln et al. 2003 | 2 | 309 | 1 | 191 | 1.24 (0.11-13.73) | |
|  | Macedo et al. 2009 | 3 | 177 | 0 | 363 | 14.34 (0.74-279.04) | |
|  | Pankratz et al. 2011 | 10 | 386 | 8 | 848 | 2.75 (1.08-7.01) | |
|  | Simon-Sanchez et al. 2008 | 3 | 269 | 4 | 271 | 0.76 (0.17-3.41) | |
|  | Sironi et al. 2008 | 3 | 135 | 0 | 50 | 2.61 (0.13-51.40) | |
|  | Wiley et al. 2004 | 2 | 99 | 0 | 45 | 2.29 (0.11-48.60) | |
|  | Lubbe et al. (IPDGC, NeuroX) | 11 | 6,541 | 4 | 5,689 | 2.39 (0.76-7.52) | |
|  | **Pooled** | **97** | **11,964** | **998** | **113,240** | **1.85 (1.38-2.50)** | |

Key: Carrier, number of samples harbouring mutation, CI, confidence intervals; CNV, copy number variant; IPDGC, International Parkinson’s disease Genomics Consortium; OR, odds ratio; *PRKN*, *Parkin* (NM_013988 and NM_004562); PD, Parkinson’s cases; PPMI, Parkinson’s Progression Markers Initiative; Reseq, Resequencing; SNV, single nucleotide variant; ^*^ German samples.

Supplementary Table 3: List of *PRKN* mutations that were considered to be pathogenic.

| Mutation |
| --- |
| PRKN ex10-12 del |
| PRKN ex10-12 dup |
| PRKN ex10 del |
| PRKN ex10 dup |
| PRKN ex11 del |
| PRKN ex11 dup |
| PRKN ex12 dup |
| PRKN ex1-4 del |
| PRKN ex1 del |
| PRKN ex1 dup |
| PRKN ex2-12 dup |
| PRKN ex2-3 del |
| PRKN ex2-3 dup |
| PRKN ex2-4 del |
| PRKN ex2-4 dup |
| PRKN ex2-4 trip |
| PRKN ex2-5 del |
| PRKN ex2 del |
| PRKN ex2 dup |
| PRKN ex2 trip |
| PRKN ex3-4 del |
| PRKN ex3-4 dup |
| PRKN ex3-5 del |
| PRKN ex3-6 del |
| PRKN ex3-7 del |
| PRKN ex3-9 del |
| PRKN ex3 del |
| PRKN ex3 dup |
| PRKN ex4-5 del |
| PRKN ex4-6 del |
| PRKN ex4-7 del |
| PRKN ex4 del |
| PRKN ex4 dup |
| PRKN ex5-12 del |
| PRKN ex5-6 del |
| PRKN ex5-7 del |
| PRKN ex5-8 dup |
| PRKN ex5-9 dup |
| PRKN ex5 del |
| PRKN ex5 dup |
| PRKN ex6-7 del |
| PRKN ex6-8 dup |
| PRKN ex6 del |
| PRKN ex6 dup |
| PRKN ex7-8 del |
| PRKN ex7-9 del |
| PRKN ex7 del |
| PRKN ex7 dup |
| PRKN ex8-10 del |
| PRKN ex8-11 del |
| PRKN ex8-9 del |
| PRKN ex8 del |
| PRKN ex8 dup |
| PRKN ex9 del |
| PRKN ex9 dup |
| PRKN partial ex4 del |
| PRKN promoter + ex1 del |
| PRKN Met1Leu |
| PRKN c.7+1G>A |
| PRKN c.8-39G>A |
| PRKN Ala31Asp |
| PRKN Arg33Stop |
| PRKN Arg33Gln |
| PRKN Gln34fs |
| PRKN Gln40Stop |
| PRKN Arg42His |
| PRKN Arg42Pro |
| PRKN Asn52fs |
| PRKN Asp53Stop |
| PRKN Val56Glu |
| PRKN Trp74fs |
| PRKN Glu79Stop  PRKN Ala82Glu |
| PRKN Pro113fs / ex3 Δ40bp |
| PRKN Pro133del |
| PRKN Lys161Asn |
| PRKN Gln171Stop |
| PRKN Gly179fs |
| PRKN Met192Leu |
| PRKN Met192Val |
| PRKN Lys211Asn |
| PRKN Cys212Gly |
| PRKN Cys212Tyr |
| PRKN Arg234Gln |
| PRKN Cys238fs |
| PRKN Thr240Arg |
| PRKN Thr240Met |
| PRKN Val258Met |
| PRKN Cys268Stop |
| PRKN Arg275Trp |
| PRKN Asp280Asn |
| PRKN Leu283Pro |
| PRKN Cys289Gly |
| PRKN Ala291fs |
| PRKN Gln311Stop |
| PRKN Gln311His |
| PRKN Cys323fs |
| PRKN Val324fs |
| PRKN Gly328Glu |
| PRKN Arg334Cys |
| PRKN Arg348fs |
| PRKN Lys349fs |
| PRKN Thr351Pro |
| PRKN Gly359Asp |
| PRKN Arg366Trp |
| PRKN Arg392fs |
| PRKN Glu395Stop |
| PRKN Ala398Thr |
| PRKN Glu409Stop |
| PRKN Thr415Asn |
| PRKN Asn428fs |
| PRKN IVS11-3C>G |
| PRKN Gly429Glu |
| PRKN Gly430Asp |
| PRKN Cys431Phe |
| PRKN Glu444Gln |
| PRKN Trp453Stop |
| PRKN Asp460fs |

Pathogenic *PRKN* (NM_013988 and NM_004562) allele are defined according to OMIM (http://omim.org/) or the Parkinson Disease Mutation Database (http://www.molgen.vib-ua.be/PDMutDB/). Key: c., complementary DNA; del, deletion; dup, duplication; ex, exon; fs, frameshift; IVS, intervening sequence; trip, triplication; Δ40bp, 40bp deletion.

Supplementary Table 4: *PRKN* variants represented on the NeuroX chip.

| Variant | Start | Stop | Reference | Alternate | PD-associated? |
| --- | --- | --- | --- | --- | --- |
| Gly430Asp | 161771240 | 161771240 | C | T | Y |
| Arg402Cys | 161781201 | 161781201 | G | A | N |
| Asp394Asp | 161781223 | 161781223 | A | G | N |
| Asp394Asn | 161781225 | 161781225 | C | T | N |
| Val380Leu | 161807855 | 161807855 | C | G | N |
| Arg366Trp | 161807897 | 161807897 | G | A | Y |
| Glu310Asp | 161990390 | 161990390 | C | G | N |
| Leu283Arg | 162206827 | 162206827 | T | C | N |
| Arg275Trp | 162206852 | 162206852 | G | A | Y |
| Phe264Leu | 162206885 | 162206885 | A | G | N |
| Arg256Cys | 162206909 | 162206909 | G | A | N |
| Cys253Tyr | 162206917 | 162206917 | C | T | N |
| Gln252Lys | 162206921 | 162206921 | G | T | N |
| Gln252* | 162206921 | 162206921 | G | A | N |
| Asp243Asn | 162394341 | 162394341 | C | T | N |
| Thr240Met | 162394349 | 162394349 | G | A | Y |
| Arg234Gln | 162394367 | 162394367 | C | T | Y |
| Leu228Leu | 162394384 | 162394384 | G | A | N |
| Ala214Val | 162394427 | 162394427 | C | A | N |
| Ala206Ser | 162475125 | 162475125 | T | A | N |
| Ser205Asn | 162475127 | 162475127 | C | T | N |
| Met192Leu | 162475167 | 162475167 | T | G | Y |
| Asn190Asn | 162475171 | 162475171 | A | G | N |
| Asp184Val | 162475190 | 162475190 | T | A | N |
| Ser167Asn | 162622197 | 162622197 | C | T | N |
| Pro153Arg | 162622239 | 162622239 | G | C | N |
| Asn144Tyr | 162622267 | 162622267 | G | A | N |
| Asp126His | 162683593 | 162683593 | C | G | N |
| Ala82Glu | 162683724 | 162683724 | G | T | Y |
| Gly77Gly | 162683738 | 162683738 | G | T | N |
| Ala46Thr | 162864377 | 162864377 | C | T | N |
| Arg42His | 162864388 | 162864388 | C | T | Y |
| Leu41Val | 162864392 | 162864392 | G | C | N |
| Leu41Leu | 162864392 | 162864392 | G | A | N |
| Gln40Gln | 162864393 | 162864393 | C | T | N |
| Pro37Leu | 162864403 | 162864403 | G | A | N |
| Gln34Arg | 162864412 | 162864412 | T | C | N |
| Asp18Asn | 162864461 | 162864461 | C | T | N |
| Glu16Asp | 162864465 | 162864465 | G | A | N |
| Exon 12 deletion | UNK | UNK | - | DEL | Y |
| Exon 8 deletion | UNK | UNK | - | DEL | Y |
| Exon 7-8 deletion | UNK | UNK | - | DEL | Y |
| Exon 7 deletion | UNK | UNK | - | DEL | Y |
| Exon 6 deletion | UNK | UNK | - | DEL | Y |
| Exon 5-6 deletion | UNK | UNK | - | DEL | Y |
| Exon 5 deletion | UNK | UNK | - | DEL | Y |
| Exon 3-6 deletion | UNK | UNK | - | DEL | Y |
| Exon 3-5 deletion | UNK | UNK | - | DEL | Y |
| Exon 3-4 deletion | UNK | UNK | - | DEL | Y |
| Exon 3 deletion | UNK | UNK | - | DEL | Y |
| Exon 2 deletion | UNK | UNK | - | DEL | Y |

Key: Alternate, alternate/observed allele; DEL, deletion; N, No; PD, Parkinson’s disease; Start, chromosome start position; Reference, reference allele; Stop, chromosomal stop position; UNK, breakpoint coordinates unknown; Y, Yes


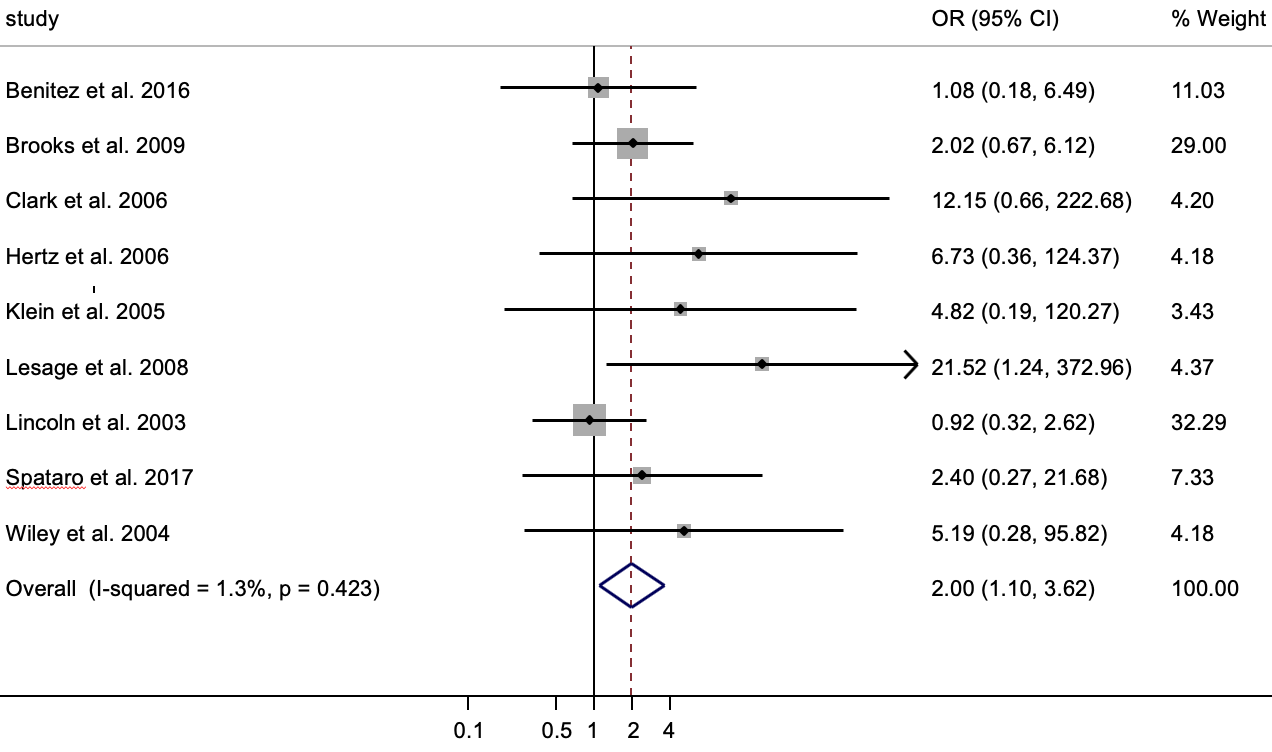


Supplementary Figure 1: Forest plot of the odds ratio (OR) of the Parkinson’s risk associated with heterozygous *PRKN* mutations using published studies that investigated biallelic *PRKN* mutations.

Boxes denote OR point estimates, their areas proportional to the inverse variance weight of the estimate. Horizontal lines represent 95% CIs. Vertical dashed line represents pooled OR point estimates. Key: CI, confidence intervals; *PRKN*, *Parkin* (NM_013988 and NM_004562); %, percentage.


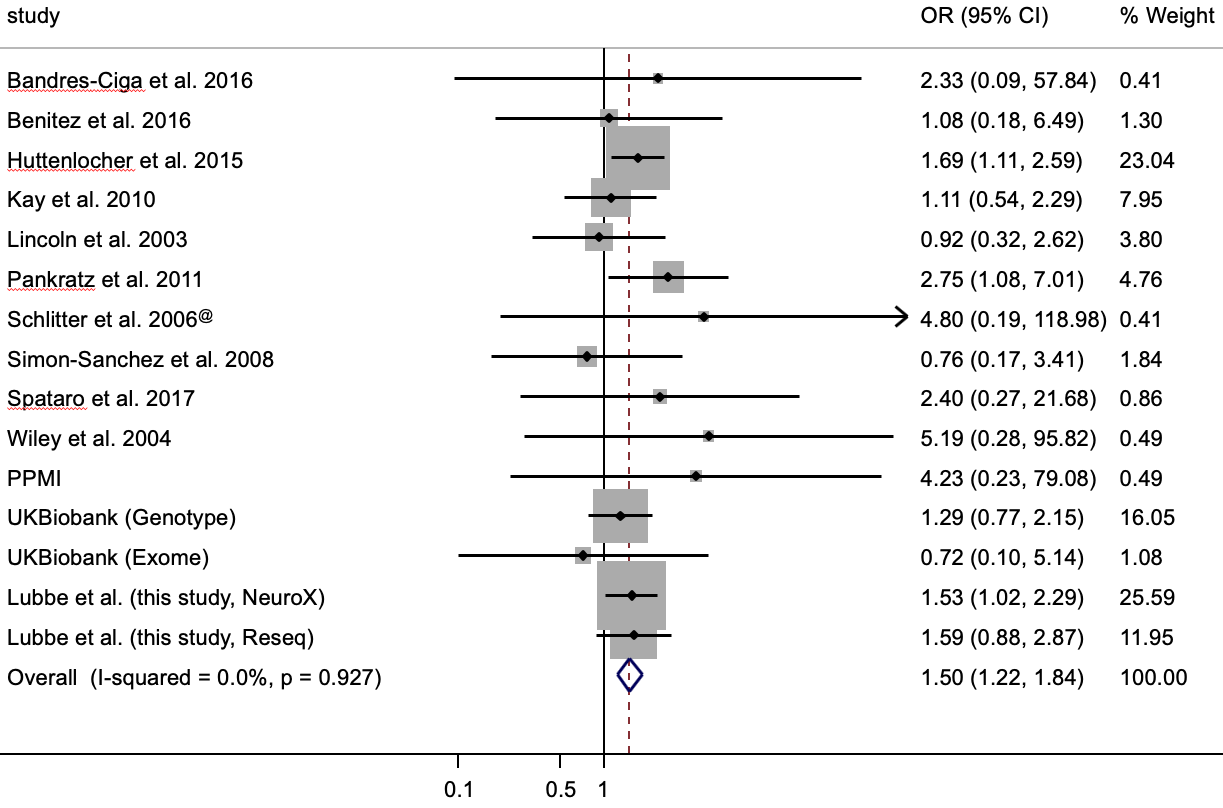


Supplementary Figure 2: Forest plot of the odds ratio (OR) of the Parkinson’s (PD) risk associated with heterozygous *PRKN* mutations, excluding studies that consisted of predominantly early-onset PD cases.

Boxes denote OR point estimates, their areas proportional to the inverse variance weight of the estimate. Horizontal lines represent 95% CIs. Vertical dashed line represents pooled OR point estimates. Key: CI, confidence intervals; IPDGC, International Parkinson’s disease Genomics Consortium; *PRKN*, *Parkin* (NM_013988 and NM_004562); %, percentage; PPMI, Parkinson’s Progression Markers Initiative; Reseq, Resequencing; ^*^German samples only.


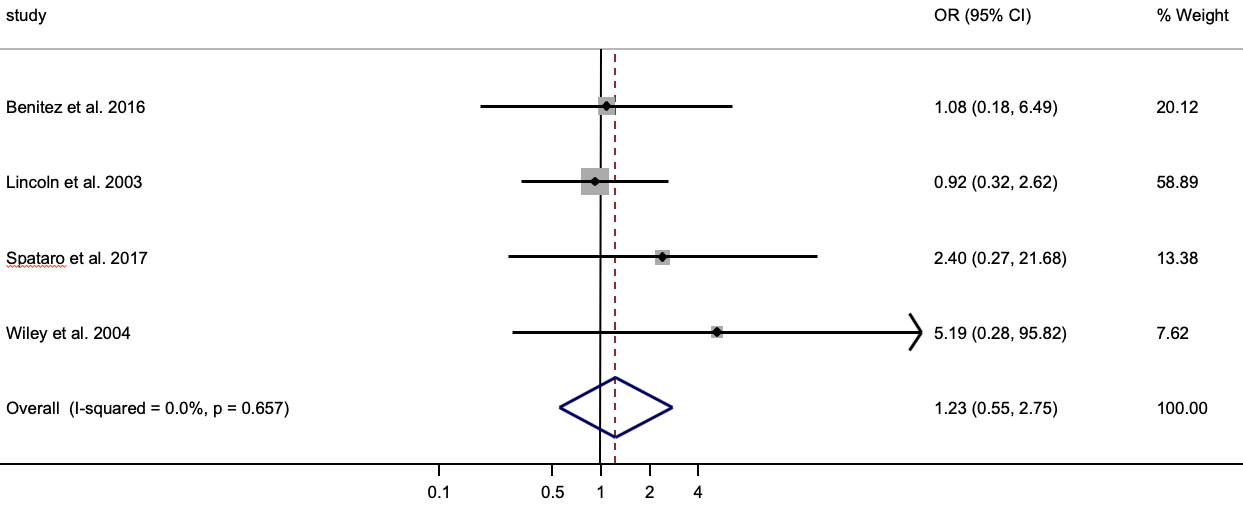


Supplementary Figure 3: Forest plot of the odds ratio (OR) of the Parkinson’s risk associated with heterozygous *PRKN* mutations, in studies that assessed biallelic mutation status and do not consist of predominantly early-onset cases.

Boxes denote OR point estimates, their areas proportional to the inverse variance weight of the estimate. Horizontal lines represent 95% CIs. Vertical dashed line represents pooled OR point estimates. Key: CI, confidence intervals; *PRKN*, *Parkin* (NM_013988 and NM_004562); %, percentage.


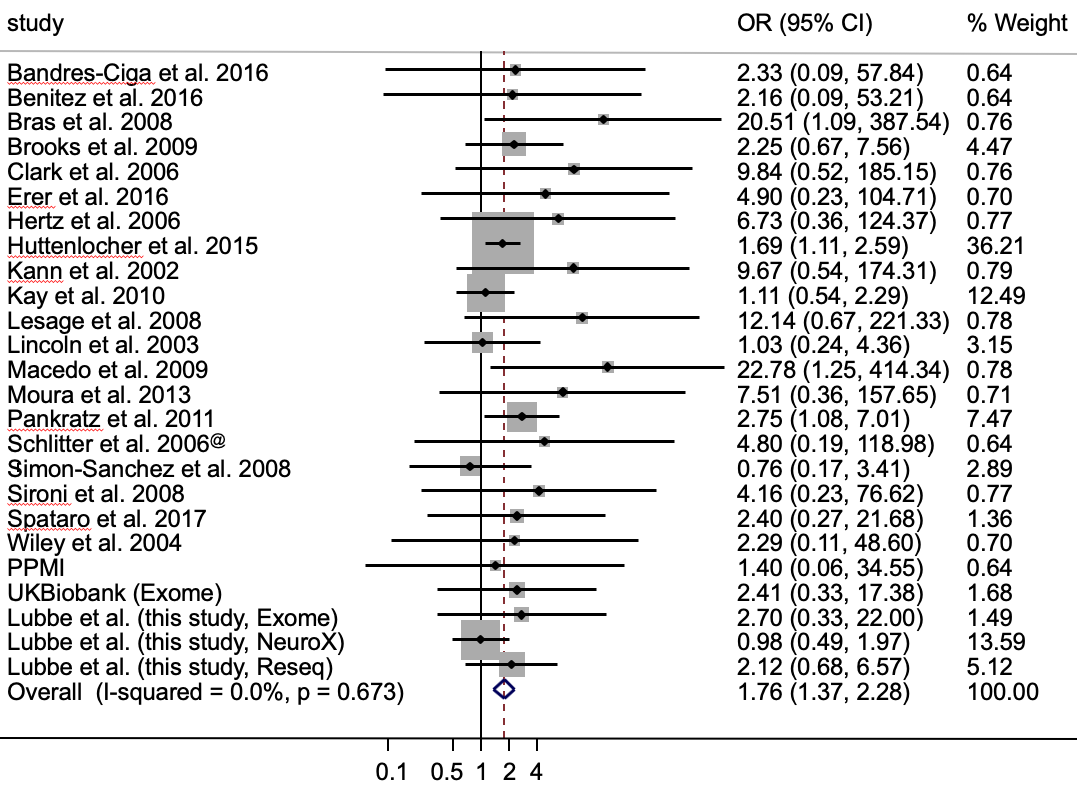


Supplementary Figure 4: Forest plot of the odds ratio (OR) of the Parkinson’s risk associated with heterozygous *PRKN* mutations, excluding p.R275W (c.823C>T, rs34424986).

Boxes denote OR point estimates, their areas proportional to the inverse variance weight of the estimate. Horizontal lines represent 95% CIs. Vertical dashed line represents pooled OR point estimates. Key: CI, confidence intervals; IPDGC, International Parkinson’s disease Genomics Consortium; p., protein reference sequence; *PRKN*, *Parkin* (NM_013988 and NM_004562); %, percentage; PPMI, Parkinson’s Progression Markers Initiative; Reseq, Resequencing; ^*^German samples only.


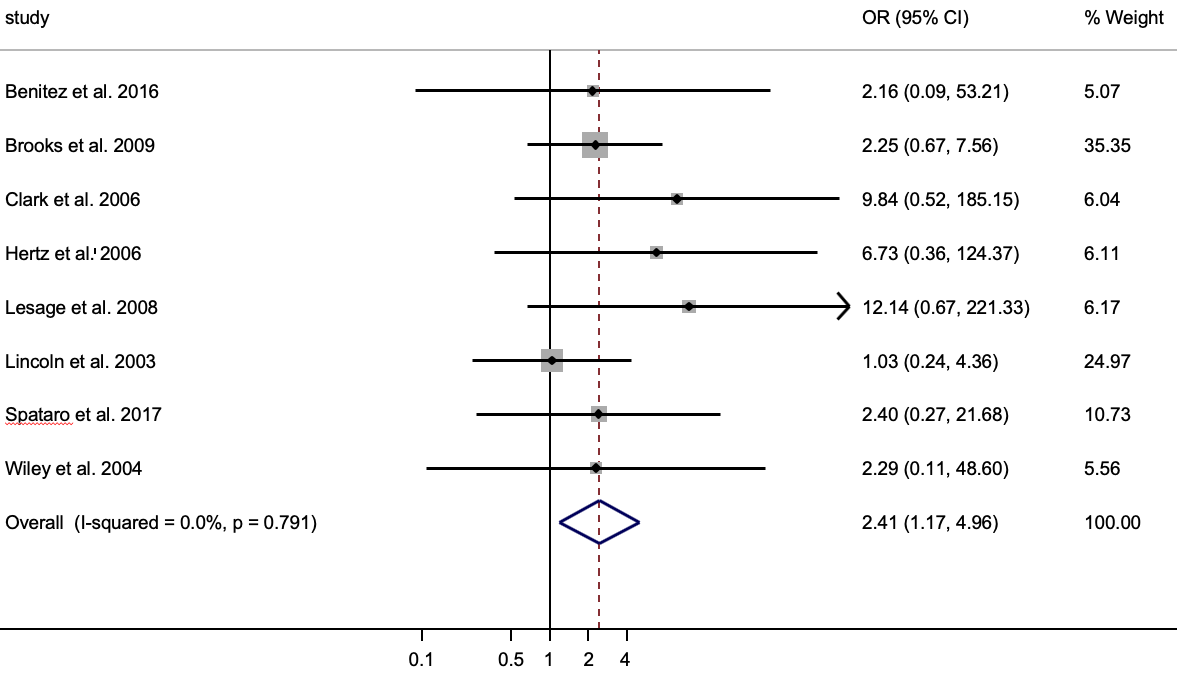


Supplementary Figure 5: Forest plot of the odds ratio (OR) of the Parkinson’s risk associated with heterozygous *PRKN* mutations, excluding R275W (c.823C>T, rs34424986), using published studies that investigated biallelic *PRKN* mutations.

Boxes denote OR point estimates, their areas proportional to the inverse variance weight of the estimate. Horizontal lines represent 95% CIs. Vertical dashed line represents pooled OR point estimates. Key: CI, confidence intervals; *PRKN*, *Parkin* (NM_013988 and NM_004562); %, percentage.
